# A qualitative study exploring approaches, barriers, and facilitators of the HIV partner notification program in Kerman, Iran

**DOI:** 10.1186/s12913-024-11049-1

**Published:** 2024-05-02

**Authors:** Fatemeh Tavakoli, Mahlagha Dehghan, Ali Akbar Haghdoost, Ali Mirzazadeh, Mohammad Mehdi Gouya, Hamid Sharifi

**Affiliations:** 1 HIV/STI Surveillance Research Center, and WHO Collaborating Center for HIV Surveillance, Institute for Futures Studies in Health, Kerman University of Medical Sciences, Kerman, Iran; 2 Reproductive Health, Family and Population Research Center, Kerman University of Medical Sciences, Kerman, Iran; 3 Institute for Global Health Sciences, University of California, San Francisco, CA USA; 4 Department of Infectious Diseases and Tropical Medicine, School of Medicine, Iran University of Medical Sciences, Tehran, Iran

**Keywords:** HIV, Notification, Counseling, Sexual partners, Iran

## Abstract

**Background:**

HIV partner notification services can help people living with HIV (PLHIV) to identify, locate, and inform their sexual and injecting partners who are exposed to HIV and refer them for proper and timely counseling and testing. To what extent these services were used by PLHIV and what are the related barriers and facilitators in southeast Iran are not known. So, this study aimed to explore HIV notification and its barriers and facilitators among PLHIV in Iran.

**Methods:**

In this qualitative study, the number of 23 participants were recruited from November 2022 to February 2023 including PLHIV (*N* = 12), sexual partners of PLHIV (*N* = 5), and staff members (*N* = 6) of a Voluntary Counseling and Testing (VCT) center in Kerman located in the southeast of Iran. Our data collection included purposive sampling to increase variation. The content analysis was conducted using the Graneheim and Lundman approach. The analysis yielded 221 (out of 322) related codes related to HIV notification, its barriers, and its facilitators. These codes were further categorized into one main category with three categories and nine sub-categories.

**Results:**

The main category was HIV notification approaches, HIV notification barriers, and facilitators. HIV notification approaches were notification through clear, and direct conversation, notification through gradual preparation and reassurance, notification due to being with PLHIV, notification through suspicious talking of the physician, and notification due to the behavior of others. Also, the barriers were classified into individual, social, and environmental, and healthcare system barriers and the facilitators were at PLHIV, healthcare staff, and community levels. Stigma was a barrier mentioned by most participants. Also, the main facilitator of HIV notification was social support, especially from the family side.

**Conclusions:**

The findings highlighted the multidimensionality of HIV notification emphasizing the importance of tailored support and education to enhance the notification process for PLHIV and their networks. Also, our results show that despite all the efforts to reduce stigma and discrimination in recent years, stigma still exists as a main obstacle to disclosing HIV status and other barriers are the product of stigma. It seems that all programs should be directed towards destigmatization.

**Supplementary Information:**

The online version contains supplementary material available at 10.1186/s12913-024-11049-1.

## Introduction

HIV notification is a process through which people living with HIV (PLHIV) are informed about their own HIV status and also take the initiative to inform their social networks, particularly their sexual and injection partners, about their HIV status [1]. HIV notification represents a complex and intricate decision-making process, characterized by numerous dilemmatic components. These include considerations such as whether to disclose or maintain confidentiality, the timing of HIV notification, the selection of the initial person to be informed, and other related factors [2]. In line with established HIV notification guidelines, there is a counseling process designed to assist PLHIV in disclosing their HIV status [3–5]. Healthcare professionals assume a pivotal role in facilitating HIV notification, employing diverse approaches that are influenced by their training and expertise. Additionally, the approach to HIV notification prevailing guidelines may vary depending on prevailing guidelines and cultural norms in specific geographic regions where HIV notification is either encouraged or discouraged. Furthermore, individual-specific factors regarding the person seeking guidance on HIV notification also significantly influence the counseling process [6].

HIV notification plays an essential role in advancing our efforts to achieve targets for HIV epidemic control. Effective counseling for PLHIV at the time of diagnosis is instrumental in facilitating their access to vital care and treatment services. Furthermore, HIV partner notification can expedite early referrals to care and promote risk reduction among high-risk discordant partners [6, 7]. It can also boost the utilization of HIV testing services by partners of newly diagnosed PLHIV, enhancing case detection and the linkage to care [8]. HIV notification represents a critical component of both prevention and treatment strategies aimed at curbing HIV transmission and optimizing the health and well-being of PLHIV [9]. Additionally, HIV notification holds the potential to contribute significantly to HIV control and prevention by promoting safer sexual practices, encouraging partner HIV testing, and fostering increased support for PLHIV [10].

However, despite the potential positive outcomes associated with HIV notification, it also entails significant risks for PLHIV. Sharing one’s HIV status with individuals who may not be accepted can leave PLHIV vulnerable to stigmatizing reactions, including social ostracism, physical harm, and workplace discrimination [9, 11]. For instance, a study conducted in sub-Saharan Africa revealed that women faced prominent barriers to HIV testing and disclosing their serostatus. These barriers included the fear of how their partners would react, the dynamics of decision-making and communication within relationships, and the attitudes of partners toward HIV testing [12]. Another study focused on PLHIV who are men who have sex with men (MSM) in Boston identified several barriers to HIV notification. These barriers included concerns about breaches of confidentiality regarding the HIV status of PLHIV and their partners, concerns about the type of sexual partner involved, potential missed sexual opportunities, and a sense of responsibility towards their partners. On the other hand, participants noted that ethical obligations, the possibility of initiating dating relationships, the timing of HIV notification, and open bidirectional communication acted as facilitators for HIV notification [13].

In Iran, HIV notification approaches are mentioned in the national HIV testing guidelines but practical modules and specific training for lay providers to successfully implement these services are lacking. The training modules need to be developed and their effects should be monitored carefully to optimize the diagnosis services [14]. New approaches that enhance the efficiency of testing and increase the coverage of treatment are needed. HIV notification is an approach that has the potential to particularly identify people with undiagnosed HIV infection who remain unlinked to prevention, treatment, and care services, and continue to be at risk of transmitting HIV vertically or through sexual and drug-injecting partners [8]. However, PLHIV and their networks in Iran may have diverse experiences with HIV notification, including HIV partner notification. To the best of our knowledge, no study has been conducted on this specific topic and approach in Iran. Conducting such a study can provide valuable insights for policymakers and service providers, enabling them to better understand the barriers and facilitators associated with HIV notification and shape effective strategies and interventions. So, this qualitative study aimed to identify the approaches, barriers, and facilitators of HIV notification, including HIV notification, among PLHIV in Iran.

## Methods

### Method and design

In this qualitative study, we used a conventional content analysis method [15] with a specific focus on three key participant groups: PLHIV, the sexual partners of PLHIV, and the staff members of the voluntary counseling and testing (VCT) center located in Kerman. Kerman is a city with a total population of about 537,000 in southeast Iran [16]. The recruitment of participants took place over the period from November 2022 to February 2023. The inclusion of PLHIV, their sexual partners, and VCT staff allowed for a comprehensive examination of the experiences, perspectives, and challenges from different views. VCT offers counseling, testing, care, and treatment for PLHIV. We carefully selected staff members from the VCT center who had prior work experience in HIV notification to PLHIV. These individuals were chosen based on their knowledge and firsthand involvement in HIV-related activities, allowing for valuable insights and informed perspectives during the study. The VCT center prioritizes protecting privacy and preventing discrimination against PLHIV through various measures. The center’s main clientele includes individuals engaged in high-risk behaviors, couples seeking HIV testing before marriage, and pregnant women, including those who are HIV-positive [17]. Services provided to PLHIV encompass addressing their basic needs, facilitating access to necessary services, maintaining ongoing communication and support, and ensuring medical care such as antiretroviral therapy (ART) under the supervision of infectious disease specialists. Additional services include psychiatric care and HIV/STI prevention measures [18].

### Sample and sampling strategy

The study was carried out by a research team specializing in HIV, who were not directly involved in providing services to the study population. Interviews were conducted by a female-trained interviewer (i.e., the first author), who visited the VCT center on specified days. Potential participants were referred by VCT staff, screened for eligibility and interest, and provided with the information necessary to obtain informed consent by the interviewer. Among the PLHIV, we started with a convenience sampling method and continued with purposive sampling. The inclusion criteria for PLHIV were the age of 18 or more and provided consent for participation in the study. Also, the staff were eligible if they had at least one year of work experience. The VCT staff members who were included if they provided verbal consent for participation. Recruitment continued during analysis until information saturation was surpassed such that no new information was found. Different demographic and occupational characteristics were also collected from the participants.

### Data collection

Our study included a total of 12 individuals living with HIV (PLHIV), five sexual partners of PLHIV (with only one partner not diagnosed with HIV), and six staff members from the voluntary counseling and testing (VCT) center. The age range of PLHIV participants was between 30 and 59 years old, while the staff members had 5.5 to 16 years of work experience in the field (Tables 1 and 2). The study reached saturation after the 19th interview, but to ensure data saturation, four more interviews were conducted. The interviews were conducted face-to-face, in Persian, using a semi-structured interview guide in a quiet, and private room with only the interviewee and the researcher present. The duration of each interview was between 30 and 35 minutes. The interview guide comprised open-ended questions according to the objectives of the study. Some of the questions were obtained from the literature review [19–22], others were obtained from local experts in the field of HIV, and some were added during the interview. All questions, including the initial and those subsequently added, were reviewed by three experts in the field. Examples of questions asked to the PLHIV group included: “How did you go about notifying others about your HIV status?”, “How did you inform your family members, friends, or acquaintances that you were infected with HIV? ”, “ What about your partner or spouse?”, “ What factors influenced your decision to notify your partner, family, and friends about your HIV status?”, and “ What barriers did you encounter, and what factors facilitated the process?”. The questions posed to the sexual partners of PLHIV were: “How did you become aware of your partner’s HIV status?” “What actions did you take after learning about your partner’s HIV status?” “How did you approach the process of notifying others about your partner’s HIV status?” and “What barriers did you encounter during this process, and what factors facilitated it?”. For the staff group, the questions asked were: “In your current practice, how do you communicate a PLHIV’s status to the individual as well as their partner, family, or friends?”, “Based on your experiences, how do PLHIV typically disclose their HIV status to their partner, family, or friends?”, and “In your experience, what are the main barriers and facilitators of HIV partner notification?” (S1). Furthermore, we incorporated follow-up questions tailored to each interviewee to gain further insights. To ensure accurate documentation, we employed a voice recorder to capture the participants’ voices during the interviews. Alongside the audio recordings, the interviewer also took supplementary notes to capture important details. The interviewers transcribed the recorded interviews verbatim and ensured the anonymity of the participants on the same day as the interview.

**Table 1:** Characteristics of people living with HIV, and their sexual partners included to assess the barriers and facilitators of HIV notification in Iran

Number	Age	Sex	Marital status	Number of children	Education level	Occupation	The socio-economic status	Duration of HIV infection
1	33	Female	Divorced	1	High school	Self-employment	Middle	9 years
2	49	Female	Married	0	High school	Labor	Low	9 years
3	39	Male	Single	0	Diploma	Chef	Low	14 years
4	46	Male	Married	2	High school	Labor	Low	11 years
5	54	Female	widow	2	Primary school	Housewife	Middle	15 years
6^a^	38	Male	Married	1	Diploma	Driver	Low	Negative
7	46	Male	Married	1	Diploma	Self-employment	Middle	6 months
8^b^	37	Female	Married	1	Bachelor	Unemployed	Middle	6 months
9	54	Female	Married	2	Diploma	Housewife	High	23 years
10^c^	59	Male	Married	2	High school	Driver	High	23 years
11	45	Male	Single	0	Primary school	Driver	Low	13 years
12	49	Female	Married	2	Middle school	Teacher	High	10 years
13^d^	54	Male	Married	2	Bachelor	Employee	High	10 years
14	42	Female	Divorced	3	Middle school	Housewife	Low	5 years
15	30	Female	Married	1	Diploma	Housewife	Middle	5 years
16^e^	43	Male	Married	1	High school	Farmer	Middle	1 year
17	37	Female	Married	2	Bachelor	Housewife	High	3 years

^a^Spouse of a PLHIV

^b^Spouse of participant number 7

^c^Spouse of participant number 9

^d^Spouse of participant number 12

^e^Spouse of participant number 15

**Table 2:** Characteristics staff of voluntary counseling and testing center included to assess the barriers and facilitators of HIV notification in Iran

Number	Age	Sex	Educational level	Position	Work experience (year)
1	39	Female	Master of Nursing	Pharmacy manager of VCT center	5.5
2	54	Female	Master of Psychology	Counselor and psychologist of VCT center	16
3	52	Male	Medical Doctor	Head of VCT center	10
4	49	Male	Bachelor	Receptionist of VCT center	6
5	44	Female	Master of Nursing	Nurse of VCT center	9
6	39	Female	Bachelor of Midwifery	Midwife of VCT center	10

### Data analysis

The collected data were systematically coded in textual form, with patterns and themes identified by one of the researchers (the first author). To ensure consistency and accuracy, the research team conducted weekly meetings to review and validate the codes and subcategories. Any ambiguities or discrepancies were thoroughly discussed and resolved during these meetings. Data analysis was performed based on the Graneheim and Lundman method [23] including (1) the interviews were transcribed verbatim (2), each of the transcriptions was considered as a unit of analysis and were read several times by the researcher to achieve a general understanding of its content (3), the sentences or entire paragraphs of text were determined as meaning units and primary codes were extracted from them (4) classifying similar preliminary codes in more comprehensive classes, and (5) determining the hidden content in the data. MAXQDA10 software was used to manage and analyze the data. Table 3 provides an overview of the analysis process performed on each text.

**Table 3:** Example of qualitative content analysis process

Meaning unit	Condensation	Code	Subcategories	Categories	Main category
When my husband found he was infected, he directly and immediately told me that I took an HIV test and it was positive *(Participant number 9).*	Notification through direct and clearly statement of spouse	Notification with normal tone	Notification through normal, clear and direct conversation	HIV notification	HIV notification, its barriers and facilitators
I do not like anyone to find out that I am a person who living with HIV, because my child is healthy, but if someone finds out, I am afraid that they will have a different view of my child *(Participant number 8).*	Worrying due to the future of child	Fears and worries	Individual barriers	Barriers of HIV notification	
There must be an experienced, patience and expert counselor in order to inform the PLHIV about the HIV status *(Participant number 20).*	An experienced, patience and expert counselor should be existed in VCTs	Presence of an experienced and patience counselor	Facilitators for PLHIV	Facilitators of HIV notification	

#### Trustworthiness

In this study, we used four criteria (credibility, transferability, dependability, and conformability) to ensure trustworthiness. These criteria were utilized to ensure rigorous and naturalistic inquiry throughout the research process [24]. To address the credibility criterion, the study site was visited before the interviews and data collection. Data credibility was also established by reviewing the adequacy of the interviews and confirming interpretations obtained from the interviews. To examine the transferability, an attempt was made to describe the characteristics of the participants in detail and consider using maximum variability in sampling. To address the dependability criterion, study processes were described to the team in detail, and the interview was audited externally; in this sense, the opinions of one foreign observer were used. For conformability, discussions in research team meetings raised additional issues that were considered for conformability. Also, for member checking, results were returned to participants to check for accuracy and resonance with their experiences.

## Results

During the interviews, 322 codes were obtained, and after removing or merging similar phrases, 221 related codes were extracted. We found one main category and three categories, including HIV notification (with five sub-categories), HIV notification barriers (with three subcategories), and HIV notification (with three subcategories) (Table 4). The category of findings explores the various approaches through which PLHIV discovered their HIV status. These approaches include learning about their HIV status from healthcare center staff, family members, sexual partners or spouses, and individuals within their social circles. Additionally, the findings examine how PLHIV disclosed their HIV status to their families, spouses or sexual partners, and the people in their networks. Also, the category addressed the barriers and facilitators of HIV notification. The results of the participants’ experiences showed that the way PLHIV informs about their condition has a significant impact on how they notify their family and friends. It was also found that there were always many barriers and problems in the way of HIV notification, which should be taken into consideration. On the other hand, there were limited facilitators for PLHIV, the staff of VCT centers, and the community, who could provide considerable help in the matter of HIV notification. Table 4 provides an overview of all subcategories and primary categories.

**Table 4:** Main categories, categories, and sub-categories extracted from content analysis

Main Category	Categories	Sub-categories
HIV notification approaches, barriers, and facilitators	1. Approaches of HIV notification	a) Notification through clear and direct conversation.
		b) Notification through gradual preparation and reassurance.
		c) Notification to family, spouse or sexual partner due to being with PLHIV at the testing time.
		d) Notification through suspicious talking of the doctor.
		e) Notification due to the behavior of others.
	6. Barriers of HIV notification	a) Individual barriers
		b) Social and environmental barriers
		c) Health care system barriers
	9. Facilitators of HIV notification	a) Facilitators for PLHIV
		b) Facilitators for Staff
		c) Facilitators for community

### HIV notification approaches

**Fig. 1:**
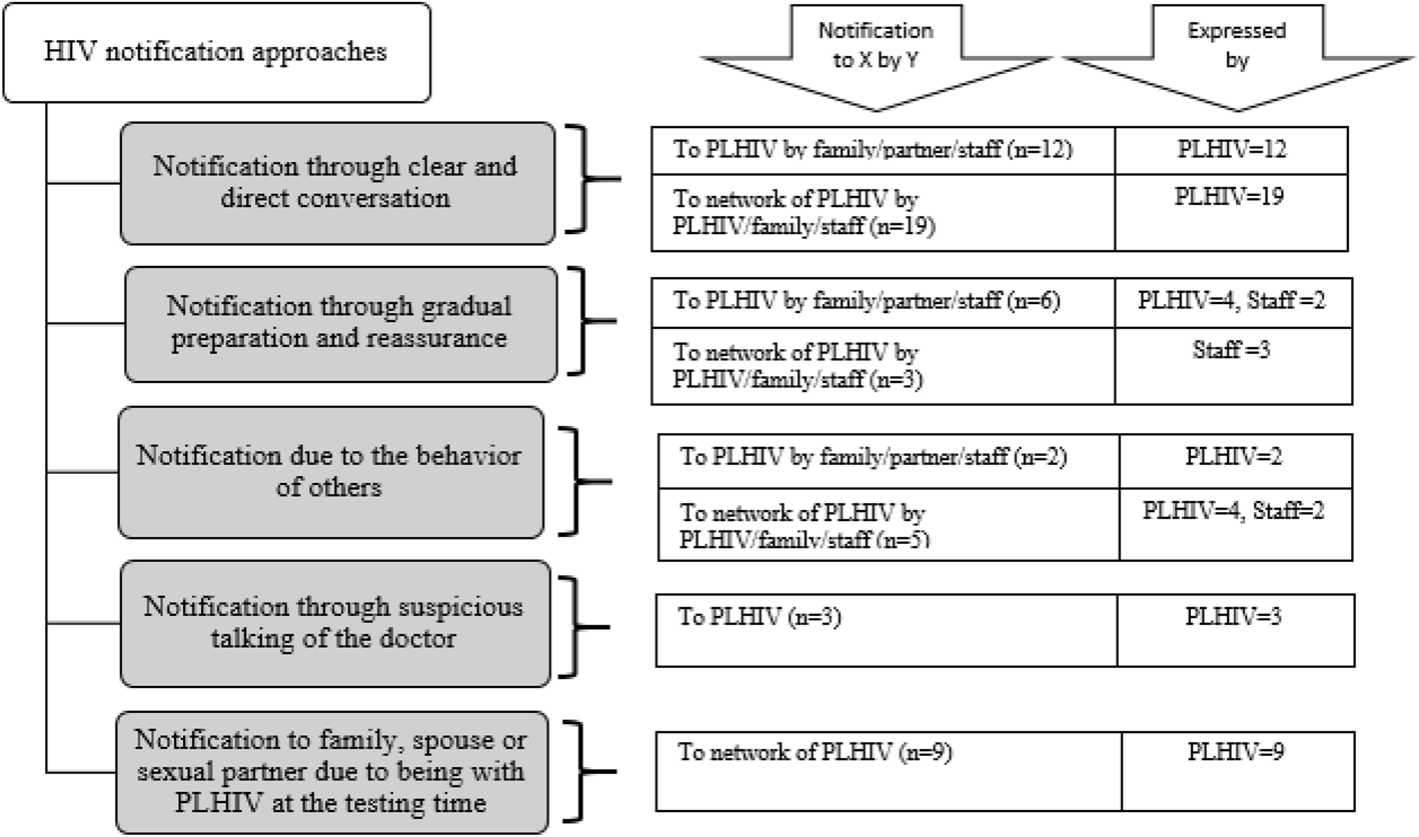
Flowchart of HIV notification approaches

We found that PLHIV were notified about their HIV status through different methods. The PLHIV also used different methods to notify the HIV status of their spouse or sexual partners, family members, friends, and other members of their networks. These approaches were placed in five subcategories, including 1) notification through clear, and direct conversation, 2) notification through gradual preparation and reassurance, 3) notification to family, spouse, or sexual partner due to being with PLHIV, 4) notification through suspicious talking of the physician, and 5) notification due to the behavior of others (Fig. 1).

#### Notification through clear, and direct conversation

According to the comments, more than half of the PLHIV had notified their spouse or sexual partner and one or more family members such as children, father, mother, sister, or brother about their condition through a clear, and direct conversation. Some patients have stated that the reason for speaking clearly and normally to their spouse or sexual partner is to be comfortable and intimate with him/her. Also, some participants stated that family members or spouses of a person living with HIV had notified the family directly and clearly.

*A 33-year-old woman, divorced, a person living with HIV said: “When I found that I was infected, I very easily told my mother and sisters that I got this disease”. (Participant number 1)*

About half of the PLHIV stated that they found out about their condition through a clear and direct conversation with the staff of VCT centers, including doctors, counselors, and laboratory staff.

Also, in one case, a person living with HIV had found that he was infected with HIV through the direct conversation of his wife (HIV negative) and his sister. It should be noted that this issue was followed up in subsequent interviews and no new data was obtained.

*A 38-year-old man, married, (HIV negative) said: “For the first time, I told my wife that your HIV test was positive, and I told my wife, just like they told me, that the disease is not dangerous and can be controlled with medicine.” (Participant number 6)*

#### Notification through gradual preparation and reassurance

Some PLHIV stated that they were notified about their HIV status gradually and received reassurance from others during the process. Similarly, the spouses or sexual partners of PLHIV were also notified gradually of their HIV status by the counselor or doctor at the VCT center.

*A 39 years old man, single, HIV positive said: “The counselor of the VCT center said in a very gentle tone and in a way that I would not get upset, and think that everything is normal, you are infected with HIV and do not worry, we are here to help you and it is like other diseases and it can be controlled.” (Participant number 3)*

#### Notification to family, spouse, or sexual partner due to being with PLHIV at the testing time

The experiences shared by some participants showed instances where the spouse and other family members, such as the sister, of a person living with HIV were notified about the individual’s HIV status. This notification occurred because these family members were present with the PLHIV at the HIV testing centers and accompanied them during the process.

*A 46-year-old man, married, and a person living with HIV, said: “The first time I went for an HIV test, the doctor called my sister who was with me and told her that I have HIV.” (Participant number 4)*

#### Notification through suspicious talking of the doctor

A small number of PLHIV stated that at first the doctor avoided informing the person and initially asked the person questions that made the person suspect that he/she was infected with HIV.

*A 46-year-old man, married, a person living with HIV said: “When I was in the hospital, the doctor asked me several questions, for example, did you have extramarital sex or not? Did you engage in risky behavior or not? I doubted that I was infected with HIV.” (Participant number 7)*

#### Notification due to the behavior of others

This category highlights that a minority of PLHIV or their families discovered their HIV infection or the infection of their relatives through various behavioral indicators. These indicators include the infected person’s frequent visits to the doctor, the use of medication, the noticeable discomfort of people around a person living with HIV, or the decision to undergo HIV testing prompted by concerns related to the individual’s behavior. In general, in this subcategory, less than half of the cases are related to notification to a person living with HIV or their spouse, and one case is related to informing the infected person’s children. It should be noted that this issue was followed up in subsequent interviews and no new data was obtained.

*A 54-year-old woman, widow, and a person living with HIV said: “I did not tell anyone myself, and my children found out step by step when they saw that I was sick and lost weight, and when they saw that I was taking these pills, they realized that I was infected with HIV.” (Participant number 5)*

### Barriers to HIV notification

The experiences of the participants indicated that HIV notification and the various methods of carrying it out are influenced by various factors, including barriers and obstacles to notification. In this study, the barriers and challenges for notification were classified into three subcategories: 1) individual barriers, 2) social and environmental barriers, and 3) healthcare system barriers.

#### Individual barriers

The majority of the participants stated that there are barriers related to the condition of a person living with HIV. This category includes five subcategories, including a) fears and worries, b) mental conditions of a person living with HIV after being informed about the infection, c) denial and non-acceptance of the infection, and d) Involvement in extramarital relationships. This subcategory mainly relates to PLHIV statements.

##### Fears and worries

The participants expressed apprehension that such HIV notification might lead to challenges or negative consequences not only for themselves but also for those in their immediate environment. The fear of disclosing their HIV status to people in their environment, fear of potential negative impacts on their children’s future, fear of social isolation and communication difficulties, fear of the health condition of the mother and the potential consequences of her being informed, as well as fears of transmitting the infection to their children, are all encompassed within this category. According to the statements of less than half of the PLHIV, these kinds of fear and worries have always been with the infected people; so, they have faced these conditions from the beginning of knowing about their condition. In a way, despite these fears and worries, PLHIV sometimes did not disclose their condition even to the closest members of their family.

*A 37-year-old woman, married, a person living with HIV said: “I did not say to my mother, because she is sick, and I do not want her to worry about me” (Participant number 17).*

##### Mental conditions of a person living with HIV after being informed about the infection

This category includes the mental state of the person due to their conditions. After they were notified about the infection, the majority of patients experienced a wide range of mental states, from hallucinations and shock to suicidal thoughts and or guilty feelings of PLHIV due to infecting people around.

*A 30-year-old woman, married, a person living with HIV said: “When I found that I was infected, I was shocked, I was hallucinating, I was thinking about everything, about death, about suicide, about the viewpoint of people around me” (participant number 15).*

##### Denial and non-acceptance of the infection

Denial and non-acceptance of the infection was another individual barrier to notifying others. PLHIV could not believe that they were infected with HIV.

*A 42-year-old woman, divorced and a person living with HIV, said: “I did not like others to know because it was hard for me to accept the disease” (participant number 14).*

##### Involvement in extramarital relationships

Two staff of the VCT center stated that one of the barriers to HIV notification is involved in extramarital relationships. It should be noted that this issue was followed up in subsequent interviews and no new data was obtained.

*A 52-year-old, married, head of a VCT Center stated: “There are some PLHIV who do not have a clear family, that is, they have a sexual partner other than their spouse, and for this reason, they do not like to disclose their HIV status.” (participant number 20)*

#### Social and environmental barriers

Another sub-category of the barriers to HIV notification for the network was social and environmental barriers. This subcategory included stigma, low HIV knowledge of society, changing the behavior of PLHIV relatives, and being easily identifiable in a small city.

##### Stigma

Stigma includes different views and perceptions towards PLHIV, notoriety, negative thoughts related to PLHIV from the viewpoint of their spouse and networks or negative view of their children, improper social image, the tendency of PLHIV families to hide HIV status from other people due to stigma in their behavior and stigmatized viewpoint toward transmission of HIV through sexual intercourse was another barrier was mentioned by most participants.

*A 54-year-old woman, married, a person living with HIV said: “The most important problem I had to tell others was that if they find out, they will look at me differently and treat me negatively” (Participant number 9).*

##### Low HIV knowledge of society

More than half of the participants stated that some people in society have little knowledge about HIV; they think that HIV can be transmitted very easily or that all PLHIV are infected because of sexual behaviors.

*A 43-year-old man, married, a person living with HIV said: “I did not tell anyone that I was infected because of lack of knowledge about PLHIV and the ways of transmission.” (Participant number 16)*

##### Changing the behavior of PLHIV relatives

According to the experiences of about half of the participants, the change in the behavior of the people around PLHIV, which includes the fear of loss and rejection, reducing the desire to meet PLHIV, decreasing or stopping sexual intercourse with the infected spouse, and inappropriate behavior of spouse and family members with PLHIV.

*A 49-year-old woman, married, a person living with HIV said: “I am a teacher and when one of my sisters found out that I was infected, she said that I will inform the education department that you are sick and you should not go to school because you are making people’s children sick. So, I preferred to not inform the rest of my network that I was infected. I believe they should act like my sister and think that this disease is contagious.” (Participant number 12)*

### Being easily identifiable in a small city

Also, a small number of participants stated that living in a small city is a barrier to HIV notification, in other words, in a small city, people can be identified, and on the other hand, the traditional atmosphere of a small city makes the PLHIV reluctant to inform the people around them. It should be noted that this issue was followed up in subsequent interviews and no new data was obtained.

*A 39-year-old woman, married and a midwife at VCT center, said: “One of the reasons why people do not inform their sexual partners about their status is they say that it is a small city and people are easily identified” (participant number 23).*

#### Healthcare system barriers

Some VCT staff believed instructions and guidelines for partner notification are not complete and they do not have enough instructions and guidelines in specific scenarios. In these situations, the consultant should decide how to notify the HIV status of some people.

*A 52-year-old, married, head of the VCT center said: “The weakness of the notification instructions is that there may be scenarios that there is no solution in practice, for example, a person may say, I cannot inform my wife, if I say, she rejects me. We cannot guarantee that you tell and there will be no problems for you” (Participant Number 20).*

### Facilitators of HIV notification

The participants identified facilitators at three levels: PLHIV, healthcare staff, and community. Facilitators of HIV notification were mostly mentioned by the staff of the VCT center.

#### For PLHIV

The participants stated that family support, the presence of an experienced and enthusiastic consultant, trust, a confidential environment, and non-judgment by the healthcare system can help the PLHIV to better and more easily explain their behavior and condition to the healthcare workers. These items also could help them to inform other people such as their spouse or sexual partner and family about their condition. Also, they stated those PLHIV who have family support are less worried about their status and they could inform other people in their networks about the infection.

*A 37-year-old woman, married, a person living with HIV said: “When I told my spouse, I am HIV infected but he is not infected with HIV, he accepted very easily and supported me” (Participant number 17).*

#### For staff

More than half of the VCT staff believed some training courses including in-person or electronically, sharing the experiences between different consultants, training courses on communication approaches, and providing practical solutions in HIV notification guidelines, can improve their ability for a more effective HIV notification.

*A 39-year-old woman, married, pharmacy of the VCT center said: “To (have a more effective) HIV notification if the educational materials can be applied in a practical way, such as educational videos, they will be more helpful.” (Participant number 18).*

#### For community

Also, more than half of the VCT staff believed some at the community level could increase the effectiveness of the HIV notification. These facilitators included education through radio and television, public awareness campaigns, culturalization in the context of HIV, and dissemination of information regarding modes of transmission to all members of society.

*A 54-year-old married, woman, consultant at the VCT center, said: “I think that general education that the whole society should be educated, and* culturalization *should be done; something that has been fixed in people’s minds, and this disease over the years that HIV is a disease that people get it due to inappropriate social behaviors, this must be changed. This is in people’s minds and this has caused fear and stigma. So, all members of the society must be aware. The media must carry out this* culturalization, *and mistake information should be corrected” (Participant number 19).*

## Discussion

Our study revealed that PLHIV in Iran received notifications about their HIV status through a variety of methods. Additionally, they employed diverse strategies to disclose their HIV status to their social networks. Notification through clear, and direct conversation and notification through gradual preparation and reassurance were mostly used for HIV notification on both sides. Also, some barriers to HIV notification were identified; fear of disclosing HIV status to the network and stigma were the main barriers to HIV notification. In addition, some facilitators like family support could smooth the HIV notification.

We found that PLHIV received notifications about their HIV status through different approaches. They also disclosed their HIV status to their networks. The majority of PLHIV received their HIV status notification and also, they disclosed their HIV status using clear and direct conversation. The result of a review indicated that HIV notification varied greatly [1]. Based on the findings of a study on methods of HIV notification by men who have sex with men, point-blank and direct disclosures have been the most commonly used strategies [25]. Also, the result of a study in Ontario mentioned that HIV notification may at first take the form of partial or indirect strategies [26]. In another study in Los Angeles and Seattle, some participants stated that directly disclosed their HIV status to sex partners at first [27]. Some studies reported that the main types of HIV notification were in a voluntary action [28, 29]. Also, in our study, some of the participants through gradual preparation and reassurance disclose HIV status. Based on WHO guidelines for HIV self-testing and partner notification, this theme is the counseling process provided by counselors that offer HIV-positive clients assistance with notifying their partners [4, 5]. It seems that HIV notification will vary in different contexts and for different people, and the health system should consider these differences and provide counseling and notification services according to each person. Also, based on our results, it seems that the way of HIV notification to PLHIV may affect how notification of them to their network will be conducted. So, these findings underscore the need for researchers to clarify more how HIV notification is implemented.

A variety of barriers impacted the HIV notification on both sides. The main barriers mentioned by the PLHIV were the fear of disclosing HIV status to the network which mostly was due to the consequences of it like losing their relationships and partnerships after they disclosed their HIV status. The findings are consistent with previous studies in other countries [30–32]. Another barrier mentioned by most participants was stigma. According to the studies conducted in Kenya, and China, the findings indicated that social stigma associated with HIV remained a prominent issue among participants and had a significant impact on their decision-making process regarding HIV status notification [33, 34]. One of the main fast-track targets of HIV control by 2025 is zero stigma and zero discrimination [35]. However, the results of different studies showed stigma, and discrimination are prevalent from different aspects toward PLHIV. To control the HIV epidemic, countries must reduce stigma and discrimination in societies. Also, to improve the process of HIV notification and use the advantages of social network testing, it is necessary to remove the obstacles to HIV notification.

Besides barriers, some facilitators were also mentioned by the participants for HIV notification. The main facilitator was social support, especially from the family side. Social and family support could help PLHIV to accept their status and explain this to their network. The same results were also explained by the Chinese PLHIV [34]. Based on the results of studies, social support is important everywhere, but in low-resource settings where services are deficient and individuals need more support from their families and communities, there are stronger incentives to disclose [36–39]. Also, the participants in our study stated that the presence of an experienced and enthusiastic consultant, trust, a confidential environment, and non-judgment by the healthcare system can facilitate notification. The result of some studies indicated that some health facilities are often ill-equipped to reassure PLHIV that they will be treated well, and health workers may not have the training to counsel patients on how to disclose their HIV status or to whom it should be disclosed [40, 41]. Trained healthcare providers, coupled with factors such as family support, can play a crucial role in facilitating HIV notification and testing, particularly when an individual expresses a desire to undergo testing alongside their partner.

Two main limitations must be considered when interpreting the findings. First, the present study was conducted in one city, and due to differences in social or religious factors in other parts of the country, the results could not be generalized to other parts of the country. Second, behavioral risk factors were self-reported by participants; therefore, the data may have been influenced by recall bias, social desirability, fear of stigma, or other negative consequences of reporting. Therefore, future studies should take into account the differences in cultural and ethnic backgrounds of Iranians when generalizing their findings.

## Conclusion

In conclusion, our study showed that there are diverse methods for HIV notification that include notification to PLHIV about their HIV status and notification to their networks. The process of HIV notification shows that the way of disclosing HIV status to PLHIV for the first time has a significant effect on notification to another network. Our findings highlight that despite the various endeavors to diminish stigma and discrimination in recent years, such as educational interventions targeted at healthcare providers, the general population, PLHIV, and key populations, stigma continues to persist as a significant barrier to disclosing one’s HIV status. Additionally, other barriers associated with HIV notification are intertwined with and influenced by the presence of stigma. In light of these findings, it is imperative that public health efforts in Iran and beyond focus on addressing barriers to HIV notification while harnessing the power of facilitators. Based on our results all programs should be directed towards destigmatization. Moreover, our study underscores the need for ongoing research to further elucidate the nuanced dynamics of HIV notification, ultimately contributing to more effective HIV control strategies and improved quality of life for PLHIV.

### Supplementary Information

**Supplementary Material 1.**

## Data Availability

Data will be available upon request submitted to the corresponding author.

## Abbreviations

PLHIV: People living with HIV
MSM: Men who have sex with men
VCT: Voluntary counseling and testing
ART: Antiretroviral therapy
