# Supplementary material for: A qualitative study exploring approaches, barriers, and facilitators of the HIV partner notification program in Kerman, Iran
Source: BMC Health Serv Res. 2024 May 2;24:570. doi: 10.1186/s12913-024-11049-1 (PMC11067232; doi:10.1186/s12913-024-11049-1)
Supplement: Supplementary file 1 — Supplementary Material 1. [file 12913_2024_11049_MOESM1_ESM.docx]

**Supplementary 1:**

**Interview guide**

Introduction:

Hello, my name is…. and thanks for the time to meet me today. I am from Kerman University of Medical Sciences, and today I will talk to you for at least 30 minutes to ask you some questions. These are the questions that help our research team to explore your opinions, views and experiences about ways to HIV notification and allow us to get information about this topic from different people's perspectives.

**Key points:**

• You can withdraw from the interview at any time.

• You cannot answer any questions that you want.

• We need to record this interview for the study, I will also take notes to help me as I speak, we will make a typed version of the interview later and at that time, your name and any other person you mention will be removed. This ensures that nothing you say can be identified.

• Any information will be provided without names attached.

1. **PLHIV group:**

**First part: Demographic information**

Age:

Gender:

Marital status:

Level of Education:

Socioeconomic status:

Job:

Duration of infection:

**Second part: views, opinions, and experiences**

1. How did you find out about your HIV status? (How did you feel? What effect did the way of telling and informing you have on your experience and feelings? What happened when you found out that you were infected? Who said? How did he/she say it?)
2. What did you do after you found out? (Ask about his lived experience of living with HIV, find out his various experiences that may have influenced his decision to inform others)
3. How did you go about notifying others about your HIV status?
4. How did you inform your family members, friends, or acquaintances that you were infected with HIV?
5. What about your partner or spouse?
6. What factors influenced your decision to notify your partner, family, and friends about your HIV status?
7. What barriers did you encounter, and what factors facilitated the process?
8. **Partners of PLHIV:**

**First part: Demographic information**

Age:

Gender:

Marital status:

Level of Education:

Socioeconomic status:

Job:

Duration of infection:

**Second part: views, opinions, and experiences**

1. How did you become aware of your partner's HIV status? (How did you feel? What effect did the way of telling and informing you have on your experience and feelings? What happened when you found out that your partner was infected? Who said? How did he/she say it?)
2. What actions did you take after learning about your partner's HIV status? (Ask about his lived experience of living with a partner who was infected with HIV, find out his various experiences that may have influenced his decision to inform others)
3. How did you approach the process of notifying others about your partner's HIV status?
4. How did you inform your family members, friends, or acquaintances that your partner was infected with HIV?
5. What factors influenced your decision to notify your family and friends about the HIV status of your partner?
6. What barriers did you encounter during this process, and what factors facilitated it?
7. **Staff group:**

**First part: Demographic information**

Age:

Gender:

Marital status:

Level of Education:

Position: Work experience (years):

**Second part: views, opinions, and experiences**

1. In your current practice, how do you communicate a PLHIV's status to the individual as well as their partner, family, or friends?
2. Based on your experiences, how do PLHIV typically disclose their HIV status to their partner, family, or friends?
3. In your experience, what are the main barriers and facilitators of HIV partner notification?
